# COVID-19 vaccination reduces mortality in patients on maintenance hemodialysis

**DOI:** 10.3389/fmed.2022.937167

**Published:** 2022-09-08

**Authors:** Leszek Tylicki, Bogdan Biedunkiewicz, Ewelina Puchalska-Reglińska, Ryszard Gellert, Michel Burnier, Jacek Wolf, Alicja Dȩbska-Ślizień

**Affiliations:** ^1^Department of Nephrology, Transplantology and Internal Medicine, Medical University of Gdansk, Gdańsk, Poland; ^2^Dialysis Unit, 7th Naval Hospital in Gdansk, Gdańsk, Poland; ^3^Department of Nephrology and Internal Medicine, Medical Center for Postgraduate Education, Warsaw, Poland; ^4^Faculty of Biology and Medicine, University of Lausanne, Lausanne, Switzerland; ^5^Translational Medicine Centre, Medical University of Gdańsk, Gdańsk, Poland

**Keywords:** maintenance dialysis, COVID-19, vaccination, incidence, fatality rate

## Abstract

Patients with chronic kidney disease on maintenance hemodialysis (HD) have a very high risk of death in the course of COVID-19. The aim of the study was to assess the effectiveness of COVID-19 vaccination to reduce the incidence of COVID-19 and the fatality rate in HD patients. A retrospective registry-based cohort study was performed in all HD adult patients in the Pomeranian Voivodeship. Vaccinations were carried out from January to April 2021 with mRNA vaccines, either BNT162b2 or mRNA-1273 with two-dose schedule. In the first analysis (2nd pandemic wave), 1,160 unvaccinated patients were included (59.7% males, 25.7% diabetic). In the second analysis (4th pandemic wave), 1,131 (59.4% male, 30.7% diabetic) individuals were included, 1,042 (92.13%) were fully vaccinated. Three hundred and fifteen HD patients (27.2%) were COVID-19 positive during the 2nd wave, and 6.9% (78/1,131) during the 4th wave. Within the fully vaccinated patients of the 4th wave, 60 were COVID-19 positive, 5.8 vs. 20.2% of unvaccinated COVID-19 positive patients in 2nd wave, respectively. COVID-19 incidence rate ratio (IRR) was 0.21 (4th wave-vaccinated vs. 2nd wave-unvaccinated) indicating a 79% reduction. The IRR between vaccinated and unvaccinated patients of the 4th wave was 0.28 in favor of vaccinated patients with 72% reduction. In the 2nd wave, 93 patients died as a result of COVID-19 (fatality rate: 29.5%). The fatality rate of fully vaccinated patients during the 4th wave was 6.7% (*p* = 0.004), while the fatality rate in the 4th wave within unvaccinated patients accounted for 11.1%. Significant clinical effectiveness of COVID-19 vaccination was demonstrated in a multicenter study in HD patients.

## Introduction

Chronic kidney disease (CKD) patients requiring maintenance hemodialysis (HD) have a very high risk of death in the course of coronavirus disease 2019 (COVID-19). Their 28-day probability of death before the start of population vaccinations was 25% for all hemodialyzed patients and 33.5% for those who required hospital-based treatment, according to a European Renal Association COVID-19 Database (ERACODA) report (1). We have reported the extremely high mortality of COVID-19-infected HD patients from the North of Poland, with a fatality rate of up to 43.8% in the oldest subjects (2). This is the result of an impaired immune response, frailty, a high burden of comorbidity and the older age of most HD patients. In addition, they have frequent personal contact in crowded areas for their in-center facility treatment. Universal and specific preventive methods to reduce the spread of the virus limited, but did not eliminate, the threat (3). HD patients have therefore been prioritized in many countries to be vaccinated against COVID-19 first (4). Unfortunately, they were not included in pivotal vaccine clinical trials, so there is not much reliable data on clinical vaccine efficacy in this population. This requires careful consideration given that immune response to vaccination may be significantly weaker in HD patients than in the general population (5).

The aim of the study was to assess the effectiveness of COVID-19 vaccination to reduce the incidence of symptomatic SARS-CoV-2 infection (COVID-19 free survival) and the fatality rate in patients on maintenance HD.

## Methods

This is a retrospective registry-based cohort study performed in all HD adult patients with chronic kidney disease in the Pomeranian Voivodeship (Northern Poland) at two time points of the COVID-19 pandemic. They were treated in 15 dialysis units (4 public and 11 private). Vaccinations against COVID-19 were carried out from January to April 2021 according to the national immunization program with mRNA vaccines, either BNT162b2 (Comirnaty, Pfizer/BionTech) or mRNA-1273 (Moderna). Vaccinations were done in two-dose schedules in accordance with the manufacturers' recommendations for the general population. In the first analysis (2nd wave of the pandemic), 1,160 unvaccinated patients were included while the second cohort of patients analyzed during the 4th wave of the pandemic included 1,131 individuals. Among the second cohort, 1,042 (92.13%) individuals were fully vaccinated. Characteristics of both cohorts are presented in the Table 1. The two groups did not differ in age and sex distribution.

**Table 1:** Characteristic of two cohorts analyzed during the second and fourth pandemic wave.

		**1st analysis**	**2nd analysis**	***p***
		**2nd wave−1.09.20–15.01.2021**	**4th wave−1.09.21–15.01.2022**	
*n*		1,160	1,131	
Sex *n* (%)	m	693 (59.7)	672 (59.4)	0.87
	f	467 (40.3)	459 (40.6)	0.87
Age years *n* (%)	<20	0	3 (0.26)	0.31
	20–44	112 (9.65)	129 (11.41)	0.17
	45–64	320 (27.59)	303 (26.79)	0.67
	65–74	399 (34.40)	377 (33.34)	0.59
	>74	329 (28.36)	319 (28.20)	0.93
Diabetes	*n* (%)	299 (25.7)	348 (30.7)	0.008
Vaccinated	*n* (%)	0	1,042 (92.13)	<0.001

The two key outcomes were analyzed in all prevalent chronically HD patients on December the 31st, 2020 (first period) and on December the 31st, 2021 (second period), and study endpoints were analyzed twice. The first analysis covered the period between September 1, 2020 and January 15, 2021 before vaccination; hence the analysis was entirely in unvaccinated patients. This period corresponded to the second wave of the pandemic in Poland, dominated by the alpha virus variant (B.1.1.7) (6). The second analysis covered the period between September 1, 2021 and January 15, 2022 after completion of basic immunizations at dialysis centers in willing patients. This period corresponded to the fourth wave of the pandemic, dominated by the delta virus variant (B.1.617.2) (7). Two subgroups of patients were analyzed during the second period: patients fully vaccinated with at least two doses of mRNA vaccine (vaccinated subgroup) and patients who had not been vaccinated (unvaccinated subgroup). Cases were considered confirmed if they had had laboratory isolation of the SARS-CoV-2 by an RT-PCR test from nasopharyngeal/oropharyngeal swabs. Testing in dialysis centers was prompted by suggestive clinical signs. The assessment of whether or not death could be linked to COVID-19 was performed by health care personnel at each center. Ethics approval for the study was obtained at the Medical University of Gdańsk (NKBBN/167/2021).

Descriptive statistics were used to outline the clinical characteristics of patients. Categorical variables were presented as relative and absolute frequency. COVID-19 incidence and COVID-19-related deaths were expressed as numbers and percentages. COVID-19 incidence rate ratio (IRR), i.e., incidence rate during 4th wave in fully vaccinated patients vs. incidence rate during 2nd wave (unvaccinated patients) was calculated. Kaplan-Meier survival curves with 95% confidence intervals were plotted to assess the probability of COVID-19-free survival. The difference between the curves was tested with a log-rank test. A Chi-square test with Yates correction for continuity was used to compare mortality rates. Data was computed with the *MedCalc*^®^ *v.20.022* statistical package (*Medcalc software Ltd*.). *P*-value of <0.05 was considered significant.

## Results

**Figure 1:**
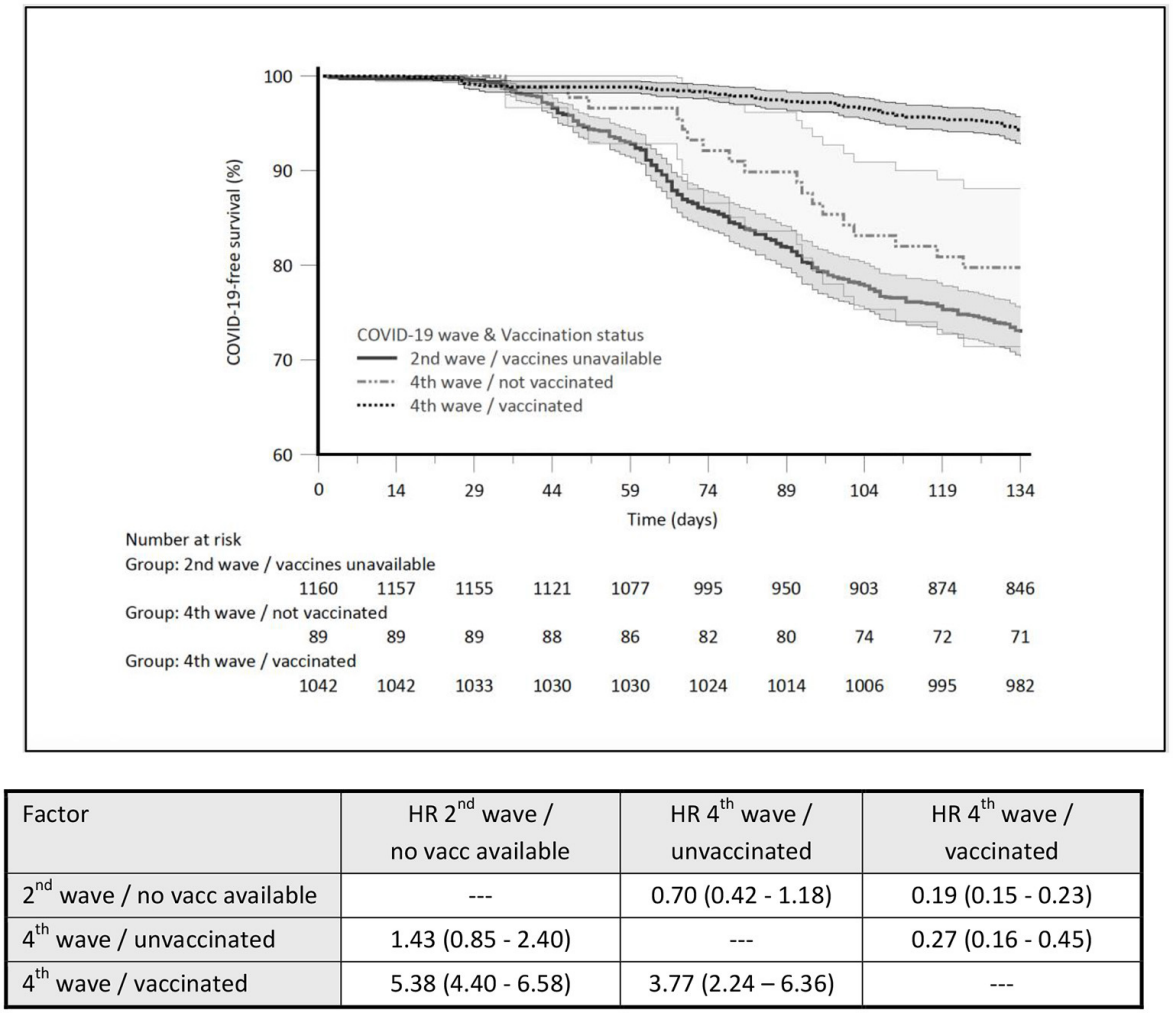
The comparison of COVID-19-free survival probability (%) between the three subgroups and the hazard ratio for COVID-19-free survival.

Three hundred and fifteen HD patients (27.2%) were COVID-19 positive during the 2nd pandemic wave, and 6.9% (78/1,131) during the 4th wave. Within the fully vaccinated subgroup of patients of the 4th pandemic wave, 60 were COVID-19 positive, which accounted for 5.8%, compared to 20.2% for unvaccinated patients. COVID-19 incidence rate ratio (IRR) was 0.21 (4th wave-vaccinated vs. 2nd wave-unvaccinated) indicating a 79% reduction of the incidence of symptomatic disease. The IRR between vaccinated and unvaccinated patients of the 4th pandemic wave was 0.28 in favor of vaccinated patients indicating a 72% reduction of the incidence of symptomatic disease. The comparison of COVID-19-free survival probability (%) between the three subgroups and the hazard ratio for COVID-19-free survival (significantly lowered by the COVID-19 vaccination) is presented in Figure 1.

During the 2nd pandemic wave, 93 patients died as a result of COVID-19 (fatality rate: 29.5%), whereas the fatality rate of fully vaccinated patients during the 4th wave was 6.7% (*p* = 0.004). The corresponding fatality rate observed during the 4th pandemic wave within unvaccinated patients accounted for 11.1%.

## Discussion

The result of the present analysis shows that COVID-19 vaccination in HD patients is effective in reducing the incidence of symptomatic disease by more than 70%. In addition, the result of the present analysis was mainly driven by a high vaccination rate in this population (on the verge of 4th pandemic wave vaccination rate in HD patients exceeded 92% in our region). Thus, the COVID-19 fatality rate in vaccinated HD patients of the fourth pandemic wave was markedly reduced as compared with the pre-vaccination period. Importantly, the improvement in the prognosis of the vaccinated group was observed while the highly contagious delta variant of the virus, causing a more severe course of COVID-19, was predominant in Poland. However, we were unable to produce direct evidence of lower fatality rate in vaccinated vs. unvaccinated in our cohorts when analysis was limited to the fourth pandemic wave, solely. There are two main factors which may explain such a phenomenon; first the vaccination rate was exceeding 92% in HD patients during the 4th pandemic wave, and second, only six COVID-19-related deaths were recorded in this period.

To the best of our knowledge, this study is the first to assess the clinical effectiveness of vaccination in a large population of HD patients, as previous studies have mainly reported on surrogate endpoints, such as antibody titers or hospitalizations (8, 9). The effectiveness in the prevention of symptomatic infection and the reduction of mortality is evident, but it seems to be lower than that observed in the general population, where it can reach more than 95% (10). This is in line with the results of previous studies in HD patients suggesting blunted immune responses to vaccination in HD patients when compared with the vaccinated general population (11). A similar humoral response in HD patients may be achieved only after the third, complementary, dose of mRNA vaccine (12). In our cohort of HD-vaccinated patients monitored during the fourth wave, only a few received a third dose of the vaccine. Given the lower antibody titer, the higher the risk of breakthrough infections (13), it is worthwhile to analyze the clinical efficacy of vaccinations in patients who received three doses of mRNA vaccine.

The main limitation of the study resulting from the registry nature of our database is the lack of information on previous SARS-CoV-2 infections. Natural immunization that may have occurred during the first pandemic waves could have significantly improved the response to vaccination and potentiated the acquired immunity of patients. Certainly, it does not undermine the effectiveness of vaccination in any way, as vaccination of previously infected patients significantly improves natural acquired immunity itself, raising it to a higher level than in convalescents (11, 14). We also do not have data on how many of the vaccinated patients received the third supplemental dose of the vaccine, which is known to improve the immune response. Secondly, although both analyzes were performed in the same dialysis centers and the two cohorts mostly overlap, they were not the same. Therefore, it cannot be excluded that the results of the study may be influenced by the fact that the patients with the greatest frailty and worst prognosis have died during the first waves of the pandemic and were no longer on dialysis during the second study period (15). Finally, due to the nature of the data register, we did not have individual patient data that would allow us to compare the characteristics of the vaccinated and non-vaccinated groups and perform any multivariable analysis.

In summary, we demonstrate the significant clinical effectiveness of COVID-19 vaccination to reduce symptomatic infections and mortality when compared to unvaccinated individuals in a multicenter study in HD patients from the North of Poland.

## Data availability statement

The raw data supporting the conclusions of this article will be made available by the authors, without undue reservation.

## Ethics statement

The studies involving human participants were reviewed and approved by Niezalezna Komisja Bioetyczna ds. Badań Naukowych at the Medical University of Gdańsk (NKBBN/167/2021). The patients/participants provided their written informed consent to participate in this study.

## Author contributions

Conceptualization: LT, BB, and AD-S. Methodology and data analysis: AD-S, LT, BB, EP-R, RG, MB, and JW. Collecting data: LT, BB, and EP-R. Writing—original draft preparation: LT, BB, and JW. Writing—review and editing: MB and AD-S. Supervision: AD-S and LT. All authors have read and agreed to the published version of the manuscript.

## Funding

This study is part of the COVID-19 in Nephrology (COViNEPH) Multicenter Observational Project registered at Medical University of Gdańsk (NKBBN/167/2021).

## Conflict of interest

The authors declare that the research was conducted in the absence of any commercial or financial relationships that could be construed as a potential conflict of interest.

## Publisher's note

All claims expressed in this article are solely those of the authors and do not necessarily represent those of their affiliated organizations, or those of the publisher, the editors and the reviewers. Any product that may be evaluated in this article, or claim that may be made by its manufacturer, is not guaranteed or endorsed by the publisher.

## References

[1] Hilbrands LB Duivenvoorden R Vart P Franssen CFM Hemmelder MH Jager KJ. COVID-19-related mortality in kidney transplant and dialysis patients: results of the ERACODA collaboration. Nephrol Dial Transplant. (2020) 35:1973–83. 10.1093/ndt/gfaa261 33151337 PMC7665620

[2] Puchalska-Reglińska E Debska-Slizien A Biedunkiewicz B Tylicki P Polewska K Rutkowski B. Extremely high mortality in COVID-19 hemodialyzed patients before the anti-SARS-CoV-2 vaccination era. Large database from the North of Poland. Pol Arch Intern Med. (2021) 131:643–8. 10.20452/pamw.16028 34105917

[3] Biedunkiewicz B Tylicki L Puchalska-Reglinska E Debska-Slizien A. Analysis of experiences in preventing COVID-19 in hemodialysis centers of the north of poland before the era of vaccination. Int J Environ Res Public Health. (2022) 19:684. 10.3390/ijerph19020684 35055503 PMC8776023

[4] Council E-E Group EW. Chronic kidney disease is a key risk factor for severe COVID-19: a call to action by the ERA-EDTA. Nephrol Dial Transplant. (2021) 36:87–94. 10.1093/ndt/gfaa314 33340043 PMC7771976

[5] Grupper A Sharon N Finn T Cohen R Israel M Agbaria A. Humoral response to the pfizer BNT162b2 vaccine in patients undergoing maintenance hemodialysis. Clin J Am Soc Nephrol. (2021) 16:1037–42. 10.2215/CJN.03500321 33824157 PMC8425628

[6] Serwin K Ossowski A Szargut M Cytacka S Urbanska A Majchrzak A. Molecular evolution and epidemiological characteristics of SARS COV-2 in (Northwestern) Poland. Viruses. (2021) 13:295. 10.3390/v13071295 34372500 PMC8310356

[7] Mazur-Panasiuk N Rabalski L Gromowski T Nowicki G Kowalski M Wydmanski W. Expansion of a SARS-CoV-2 delta variant with an 872 nt deletion encompassing ORF7a, ORF7b and ORF8, Poland, July to August 2021. Euro Surveill. (2021) 26:902. 10.2807/1560-7917.ES.2021.26.39.2100902 34596017 PMC8485581

[8] Bouwmans P Messchendorp AL Sanders JS Hilbrands L Reinders MEJ Vart P. Long-term efficacy and safety of SARS-CoV-2 vaccination in patients with chronic kidney disease, on dialysis or after kidney transplantation: a national prospective observational cohort study. BMC Nephrol. (2022) 23:55. 10.1186/s12882-022-02680-3 35123437 PMC8817171

[9] El Karoui K Hourmant M Ayav C Glowacki F Couchoud C Lapidus N. Vaccination and COVID-19 dynamics in dialysis patients. Clin J Am Soc Nephrol. (2022) 17:395–402. 10.2215/CJN.10300721 35144970 PMC8975027

[10] Polack FP Thomas SJ Kitchin N Absalon J Gurtman A Lockhart S. Safety and efficacy of the BNT162b2 mRNA Covid-19 vaccine. N Engl J Med. (2020) 383:2603–15. 10.1056/NEJMoa2034577 33301246 PMC7745181

[11] Callaway E. COVID super-immunity: one of the pandemic's great puzzles. Nature. (2021) 598:393–4. 10.1038/d41586-021-02795-x 34650244

[12] Biedunkiewicz B Tylicki L Slizień W Lichodziejewska-Niemierko M D chodzie M. Waning humoral response after COVID-19 mRNA vaccination in maintenance dialysis patients and recovery after a complementary third dose. Vaccines. (2022) 10:433. 10.3390/vaccines10030433 35335065 PMC8950255

[13] Piano Mortari E Russo C Vinci MR Terreri S Fernandez Salinas A Piccioni L. Highly specific memory B cells generation after the 2nd dose of BNT162b2 vaccine compensate for the decline of serum antibodies and absence of mucosal IgA. Cells. (2021) 10:541. 10.3390/cells10102541 34685521 PMC8533837

[14] Tylicki L Biedunkiewicz B Dabrowska M Slizien W Tylicki P Polewska K. Humoral response to SARS-CoV-2 vaccination promises to improve the catastrophic prognosis of hemodialysis patients as a result of COVID-19: the COViNEPH Project. Pol Arch Intern Med. (2021) 131:797–801. 10.20452/pamw.16069 34351091

[15] Tylicki L Puchalska-Reglińska E Tylicki P Och A Polewska K Biedunkiewicz B. Predictors of mortality in hemodialyzed patients after SARS-CoV-2 infection. J Clin Med. (2022) 11:285. 10.3390/jcm11020285 35053983 PMC8778392

